# Performance and reliability improvement in intercalated MLGNR interconnects using optimized aspect ratio

**DOI:** 10.1038/s41598-022-05222-x

**Published:** 2022-01-27

**Authors:** Bhawana Kumari, Rohit Sharma, Manodipan Sahoo

**Affiliations:** 1 Department of Electronics and Communication Engineering, Indian Institute of Technology (Indian School of Mines), Dhanbad, 826004 India; 2 Department of Electrical Engineering, Indian Institute of Technology Ropar, Rupnagar, Punjab India

**Keywords:** Engineering, Electrical and electronic engineering

## Abstract

In this work, aspect ratio of various intercalation doped MLGNR interconnects are optimized using a numerical approach to achieve improved performance and reliability. A numerical optimization method is presented to estimate optimized aspect ratio considering combined effects of performance, noise and reliability metrics for any arbitrary nano interconnect system. This approach is cost effective and will be extremely useful to industry for selection of aspect ratio of interconnects as it is a non-SPICE method and reduces fabrication iterations for achieving desired performance and reliability. Our numerical method suggests that by minimizing the figure of merit (i.e. Noise Delay Power Product / Breakdown Power $P_{BD}$ ratio), aspect ratio of FeCl_3_ doped MLGNR interconnect is optimized at 0.987, 0.61 and 0.579 for local, intermediate and global level, respectively at 7 nm node. Comparing the optimized performance metrics in this work with the estimated metrics at prescribed aspect ratio by *IRDS* roadmap, delay, noise delay product (*NDP*), power delay product (*PDP*), $PDP/ P_{BD}$ ratio and figure of merit are improved by ($\sim$2% and $\sim$25%), ($\sim$44% and $\sim$50%), ($\sim$9% and $\sim$48%), ($\sim$6% and $\sim$48%) and ($\sim$49% and $\sim$68%) for 10 $\mu$ m and 1 mm long Fecl_3_ doped MLGNR interconnect, respectively at 7 nm node. Increase in contact resistance leads to significant decrease in performance and increase in optimized aspect ratio of local Fecl_3_ doped MLGNR interconnect. Scaling down from 10 to 7 nm node results in increase of optimized aspect ratio in all levels of interconnects. Even though the performance of MLGNR degrades with scaling down but when compared to copper, the performance improves with technology scaling. Finally, this study provides circuit designers a detailed guideline for selecting an optimized aspect ratio for achieving better performance, power efficiency and reliability in doped MLGNR interconnects.

## Introduction

Copper interconnects have reached their performance limits due to high resistivity, grain boundary scattering effects and electromigration issues^1^. Their current carrying capacity has reduced resulting in poor IC performance^2,3^. Other alternative materials such as cobalt and ruthenium were proposed due to their higher EM reliability even though they have higher resistivity than copper^4,5^. Graphene nanoribbons (*GNRs*) have proved to have high conductivity, great electromigration reliability and superior transport properties making them more suitable as an alternative interconnect material than copper^6,7^.

Multilayer Graphene Nanoribbons (*MLGNRs*) are preferred over single layer graphene because of their lower resistivity. However, due to inter-sheet electron hopping, it suffers from decrease in conductivity. To solve this issue, intercalation doped *MLGNRs* were first proposed by Xu et al.^6^. Sahoo et al. analyzed the crosstalk and reliability effects in MLGNR interconnects in^8^. Liao et al. in^9^ investigated high field transport in GNRs up to breakdown. In^10^, Jiang et al. proposed Fecl_3_ doped MLGNR for better performance and reliability to challenge copper as an interconnect. They showed that Fecl_3_ doping is very efficient for diffusion in scaled MLGNRs, is stable at room temperature and shows excellent current carrying capacity >200 MA/Cm^2^. But Jiang et al. did not consider the effects of via and aggressor nets which is a pressing concern in IC design. Agashiwal et al. in^11^ engineered a CMOS-compatible solid-phase growth technique to yield large-area multilayer graphene on dielectric (SiO) and metal (Cu) substrates and subsequently demonstrating multi-level interconnects with metal vias. Also, Fischer and his team demonstrated an ingenious method in^12^ to produce metallic GNRs based on the atomically precise bottom-up synthesis. These fabricational advancements have strengthened the claim of graphene nanoribbons as an effective alternative to commercial metals. Wang et al. in^13^ also advertised graphene nanoribbon as a promising candidate for quantum electronic applications praising its high mobility and current-carrying capability. Nishad et al. in^14^ optimized thickness of Lithium and AsF_5_ intercalated Top-Contact MLGNR (TC-MLGNR) interconnects and compared with copper and pristine interconnects. Both Jiang et al. and Nishad et al. have not shown any dimensional optimization for improved performance and reliability issues which is a concerning factor to consider for commercialization of MLGNR interconnects in near-future VLSI circuits.

In this work, a numerical model is developed for optimization of aspect ratio (*AR*) by minimizing delay and FOM ($NPDP/P_{BD}$ ratio) for local, intermediate and global level MLGNR interconnects considering different intercalation dopants. This model is supported by the simulation results provided in “Results and discussion” section . Delay, Delay/$P_{BD}$, *NDP* and *PDP*, $PDP/P_{BD}$ and $NPDP/P_{BD}$ are compared by considering *AR* prescribed by IRDS 2018 roadmap^1^ and the optimized *AR* obtained from this work (for $FeCl_3$ doped MLGNR). Impact of scaling on optimization of *AR* is studied for two representative nodes, 7 *nm* and 10 *nm*. Effect of contact resistance on numerically optimized *AR* is shown which acts as an important factor in sub-10 nm technology nodes. Our study is in accordance with the trends observed in IRDS roadmap. A realistic model including the effects of crosstalk and vias is adopted which is not considered in^10^. Modeling of coupled three conductor line system shown in Fig. 3 is performed in Verilog-A. This proposed numerical methodology is applicable to all types of nano-interconnects making it a generalized model. We have validated this model with experimental data from^10^ and simulation data from^14^.

The remainder of the paper is organized as follows: “Circuit modeling of MLGNR interconnects—an overview” section presents the equivalent electrical model of MLGNR interconnects. “Formulation and methodology” section proposes an numerical model for optimizing *AR*. “Results and discussion” section presents the simulation results. Finally, “Conclusion” section concludes this paper.

**Figure 1:**
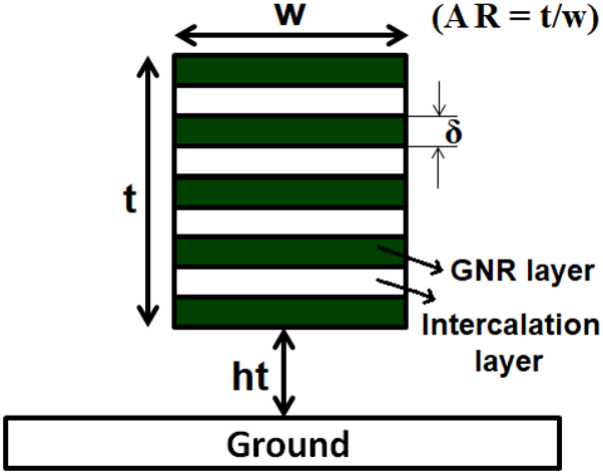
Structural representation of doped MLGNR interconnect.

## Circuit modeling of MLGNR interconnects—an overview

**Figure 2:**
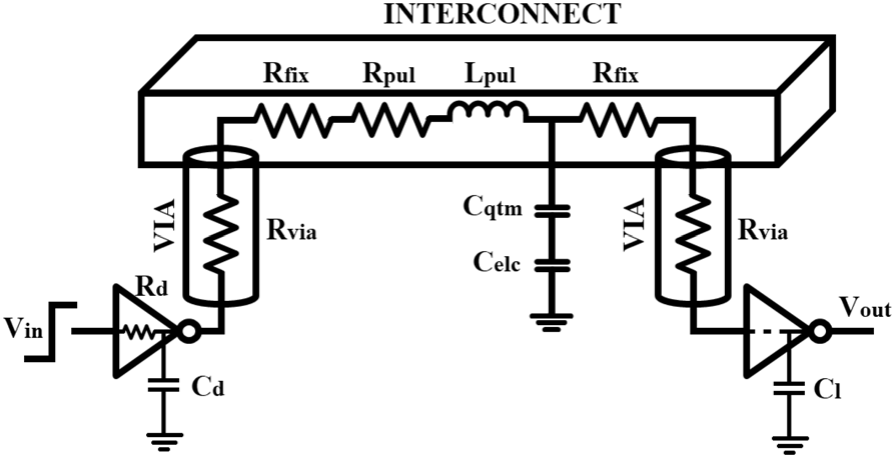
Electrical equivalent of MLGNR interconnect system.

The structural representation of MLGNR interconnect is shown in Fig. 1. Here, thickness and width are denoted by *t* and *w*, respectively and *ht* denotes the height of interconnect above ground plane. The spacing between two layers of MLGNR is represented by $\delta$. The advantage of doping is that each layer of intercalated MLGNR can be understood as stacked single layer *GNR*s because these layers do not have any interaction with each other. So, every layer of MLGNR can be modeled as Equivalent Single Conductor (*ESC*) model as shown in Fig. 2.

Electrical equivalent model of MLGNR interconnect system is shown in Fig. 2 where driver and load (at active device level) are connected to the interconnect metal line though vias. Copper is chosen as the via material in this study whose dimensions are taken from IRDS 2018 roadmap.

The electrical circuit of MLGNR interconnect model consists of lumped resistance, ($R_{fix}$ = $\frac{R_{con}+R_{qtm}}{2}$) where $R_{con}$ is the imperfect contact resistance (MLGNR to Nickel contact) which is taken as 6 $\Omega -\mu m$/W^10^. $R_{qtm}$ is the quantum resistance which is given by^8^,

1 $$\begin{aligned} R_{qtm}=\frac{\frac{h}{2q^{2}}}{N_{ch}N_{oL}}=\frac{12.94K \Omega }{N_{ch}N_{oL}} \end{aligned}$$

where $N_{oL}$ denotes the total number of layers present in MLGNR and and $N_{ch}$ represents number of conducting channels associated with each layer of MLGNR^8^.

The per unit length (p.u.l) distributed resistance of MLGNR as shown in Fig. 2 can be calculated as^6^,

2 $$\begin{aligned} R_{pul}=\frac{1}{G_{pul}*N_{oL}} \end{aligned}$$

where $G_{pul}$ represents the p.u.l conductance of a single layer MLGNR as expressed below^6^,

3 $$\begin{aligned} G_{pul}=\frac{2q^2}{h}f(\lambda _{D},w) \frac{2w^2}{h\nu _{f}} 2K_bTlog\left( 2cosh\left( \frac{E_{f}}{2K_bT}\right) \right) \end{aligned}$$

where *q* is the elementary charge, *h* is Planck’s constant, $\nu _f$ = $10^6$ m/s is the Fermi velocity, $E_f$ is the Fermi level, $K_b$ is Boltzmann’s constant, *T* is the temperature (here room temperature is considered), *w* is the width of the MLGNR and $f (i,\lambda _{D},w)$ as expressed in^10^, is a function of specularity parameter where, $i=(1-P)$, $\lambda _{D}$ is the mean free path determined by the Matthiessen’s equation^10^. *P* represents specularity index which is a measure of specularity of GNR edges. $P=1$ means completely specular edges whereas $P=0$ implies completely diffusive edges^6^.

The p.u.l capacitance ($C_{pul}$) is a series combination of quantum capacitance ($C_{qtm}$) and electrostatic capacitance ($C_{elc}$) as described below^15^.

4 $$\begin{aligned}&C_{qtm}=N_{ch}N_{oL}.\frac{4q^{2}}{h\nu _{F}}=N_{ch}N_{oL}\times 193.18aF/\mu m \end{aligned}$$

5 $$\begin{aligned}&C_{elc}=C_{gnd}+C_{inter}+C_{intra} \end{aligned}$$

where $C_{gnd}$, $C_{inter}$ and $C_{intra}$ are wire to ground capacitance, inter-layer capacitance and intralayer capacitance, respectively explained in detail in^15^.

**Figure 3:**
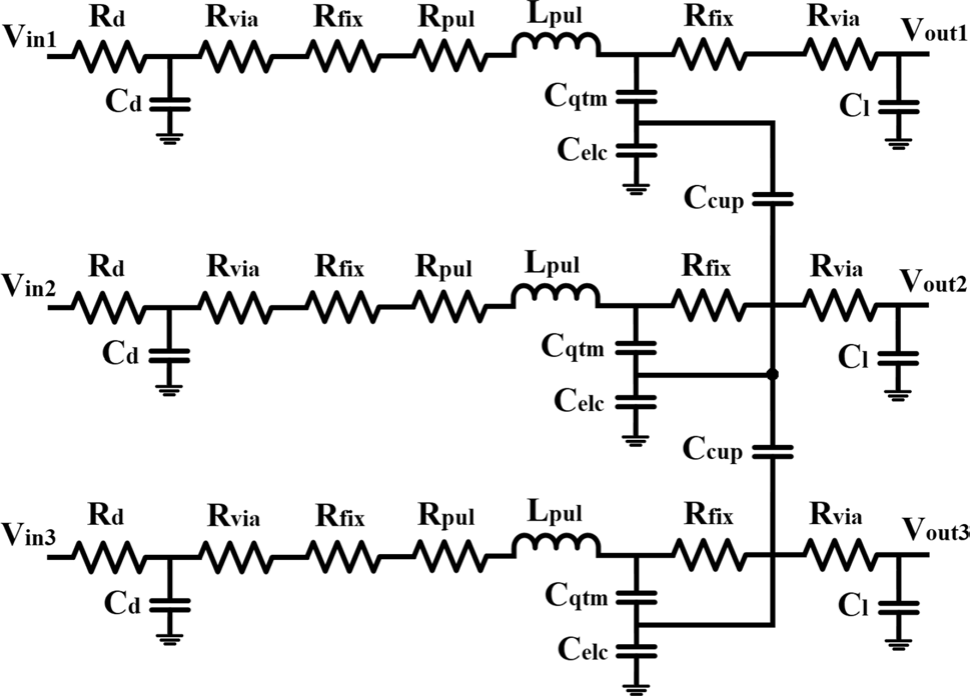
Electrical equivalent of three-line MLGNR interconnect system.

The coupling capacitance between two interconnect lines as shown in Fig. 3 is given by^8^,

6 $$\begin{aligned} C_{cup}=\epsilon _0\epsilon _r\Biggl [1.14\frac{t}{S} \Bigl (\frac{ht}{ht+2.06S}\Bigr )^{0.09}+0.74 \Bigl (\frac{w}{w+1.59S}\Bigr )^{1.14}+1.16\Bigl (\frac{w}{w+1.87S} \Bigr )^{0.16}\Bigl (\frac{ht}{ht+0.98S}\Bigr )^{1.18}\Biggr ] \end{aligned}$$

where $\epsilon _0$ and $\epsilon _r$ are the dielectric constant and permittivity in the free space, respectively. *t* denotes the thickness of MLGNR, *ht* represents the height of the interconnect above ground plane and *S* is spacing between adjacent interconnects.

The p.u.l inductance ($L_{pul}$) of MLGNR is sum of p.u.l kinetic inductance ($L_{kn}$) and self inductance ($L_{sf}$) and is expressed as $L_{pul}$ $\approx$ $(L_{kn} + L_{sf})$^15^. $L_{sf}$ and the electrostatic capacitance ($C_{elc}$) of MLGNR are considered same as of copper interconnect having equal dimensions^10^. Here, we have ignored the mutual inductance because the analysis is done for low/mid frequency range where effect of mutual inductance is negligible.

The capacitance model used for copper and cobalt interconnect is taken from^8^ and the resistance model is described in^4^ where 30% of the copper line is occupied by liner and cobalt has no liner.

## Results and discussion

**Table 1:** Electronic properties of different intercalated MLGNR interconnects.

Properties	Neutral^6^	AsF_5_^22^	FeCl_3_^10,22^	Lithium^14,23^
Stage of intercalation	NA	Stage 1	Stage 2	Stage 1
Mean free path $(\mu m)$	0.42	1.03	1	1.76
Fermi level (eV)	0.2	0.6	0.68	1.5
Avg layer Spacing (nm)	0.34	0.575	0.47	0.37

The simulations are carried out in Cadence Virtuoso, version IC 6.1.6-64B.5004 under standard desktop environment. The coupled three conductor model as described in Fig. 3 is modeled using Verilog-A including the effects of vias ad crosstalk. IRDS 2018 roadmap^1^ is considered for extracting the parameters used in the calculations. 7 *nm* technology node is used for intermediate and global level interconnects.

Length of local level (Metal line 1), intermediate level (Metal level 2) and global level (Metal level 6) interconnects are considered as 500 *nm*, 10 $\mu m$ and 1 *mm*, respectively. Nearly specular (i.e. $P=0.8$) MLGNR interconnects is considered for all the calculations. Properties of various intercalated MLGNR interconnect materials are described in Table 1.

Aspect ratio is varied from 0.4 to 3.2 for intermediate level and 0.4 to 3.4 for global level interconnects. Width of local level (Metal line 1) interconnects is considered as $w_{min}$ as specified in IRDS roadmap^1^ for 7 and 10 nm nodes. It is taken as 1.5 times the $w_{min}$ for intermediate level and 5 times the $w_{min}$ for global level as specified in IRDS roadmap^1^ to reduce delay and power consumption^24^.

### Optimizing AR by minimizing various metrics

**Figure 4:**
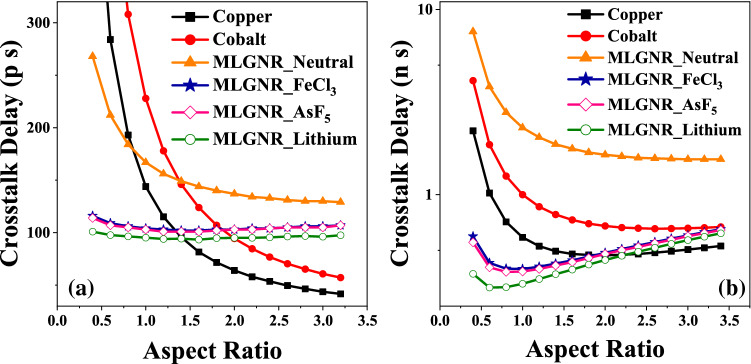
Optimized *AR* at minimum crosstalk induced delay in (**a**) intermediate level (L = 10 $\mu$m) and (**b**) global level (L = 1 mm) interconnects.

Figure 4 shows the optimized *AR* in AsF_5_, Fecl_3_, Lithium doped MLGNR, neutral MLGNR, cobalt and copper interconnects. Intercalated MLGNR experiences very low delay when compared to neutral MLGNR and conventional metals irrespective of interconnect length for smaller *AR*. The *AR* for intermediate level, $AsF_5$, $FeCl_3$, *Lithium* doped MLGNR optimizes at 1.4, 1.4 and 1.2 for intermediate level and at 1.0, 1.0 and 0.8 for global level interconnects. Delay in neutral MLGNR and copper interconnect saturates for higher *AR*. Also it can be observed that copper and cobalt interconnects outperform all the doped MLGNRs after reaching an *AR* value of 1.4 and 1.8, respectively for intermediate level. Also, copper performs better than Fecl_3_ doped MLGNR for an *AR* value of 2.0 or higher for global level interconnects. *AR* of global level interconnects optimizes at larger value as compared to intermediate level because of higher resistance as implied by Fig. 14 and this trend matches with the IRDS suggestion.

**Figure 5:**
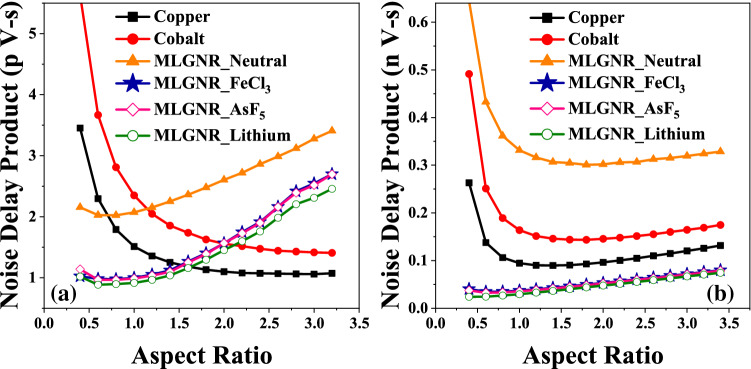
Optimized *AR* at minimum noise delay product in (**a**) intermediate level (L = 10 $\mu$m) and (**b**) global level (L = 1 mm) interconnects.

Optimized *AR* at minimum *NDP* is shown in Fig. 5. AsF_5_, Fecl_3_, Lithium doped MLGNR is optimized at 0.8, 0.8 and 0.6 for intermediate level and at 0.6 for global level interconnects. Intermediate level copper and cobalt cut all the doped MLGNR at *AR* value of 1.6 and 2, respectively. Hence, intermediate level MLGNR interconnects are more prone to noise for higher *AR* as compared with copper and cobalt as shown in Fig. 5a. But Fig. 5b shows that this is not the case for global level interconnect.

**Figure 6:**
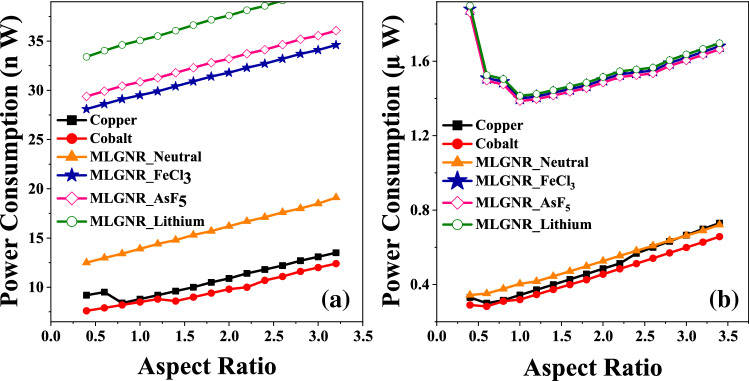
Comparison of power consumption in interconnects at (**a**) intermediate level (L = 10 $\mu$m) and (**b**) global level (L = 1 mm) interconnects.

**Figure 7:**
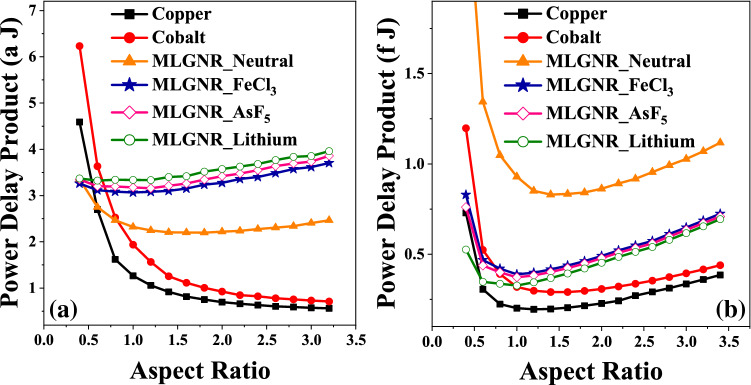
Optimized *AR* at minimum power delay product in (**a**) intermediate level (L = 10 $\mu$m) and (**b**) global level (L = 1 mm) interconnects.

Figure 6a,b show power consumption in cobalt, copper, neutral and various doped MLGNR interconnects for intermediate and global level respectively. Switching power is the dominating factor in total power consumed by the repeaters. $P_{switch}$ increases as *AR* increases. So, interconnects with large *AR* consume more power as inferred from Fig. 6. Cobalt interconnect consumes least power among others as it has lower capacitance and higher resistance value.

The least *PDP* is obtained at *AR* value of 1.0, 1.0 and 0.6 for intermediate level AsF_5_, Fecl_3_, Lithium doped MLGNR interconnects but copper and cobalt beats them at even lower *AR* as shown in Fig. 7a. Figure 7b shows optimization at 0.8 which is also the crossing point after which copper exceeds all the doped MLGNR interconnect.

**Figure 8:**
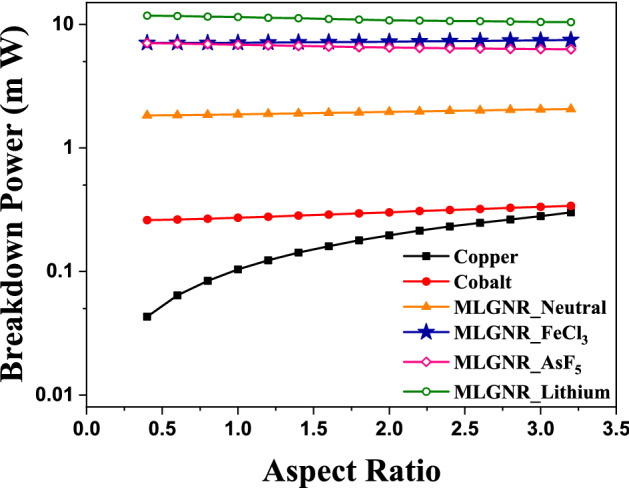
Variation of breakdown power versus aspect ratio for 10 $\mu$m long interconnects.

**Figure 9:**
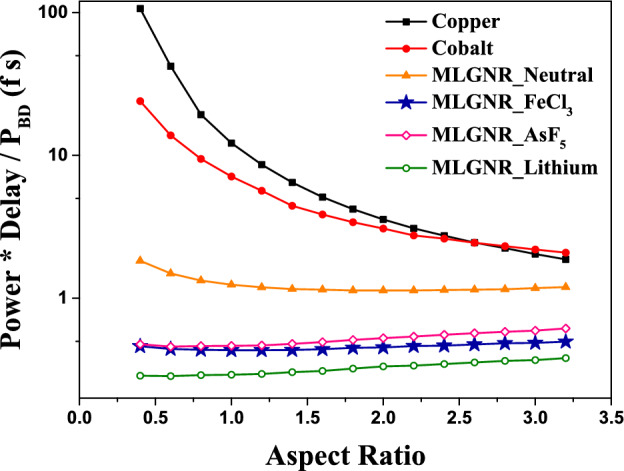
Optimized *AR* at minimum (power delay product/breakdown power) for 10 $\mu$m long interconnects.

Figure 8 shows the variation of breakdown power with respect to aspect ratio of interconnects. Here, we can see an increasing curve of breakdown power as the aspect ratio increases. Thermal healing length increases with an increase in *AR*. Breakdown power is a function of thermal healing length for intermediate level when $L_{TH}$ is comparable to GNR length. doped MLGNRs appear to be the most reliable candidates among all as they have large breakdown power.

The optimization of *AR* by minimizing the metric $(Power*Delay/P_{BD})$ is shown in Fig. 9. The optimization for AsF_5_, Fecl_3_, Lithium doped MLGNR interconnects are obtained at 1.0, 1.0 and 0.6. In case of cobalt and copper interconnects, the metric decreases with increasing *AR*.

**Figure 10:**
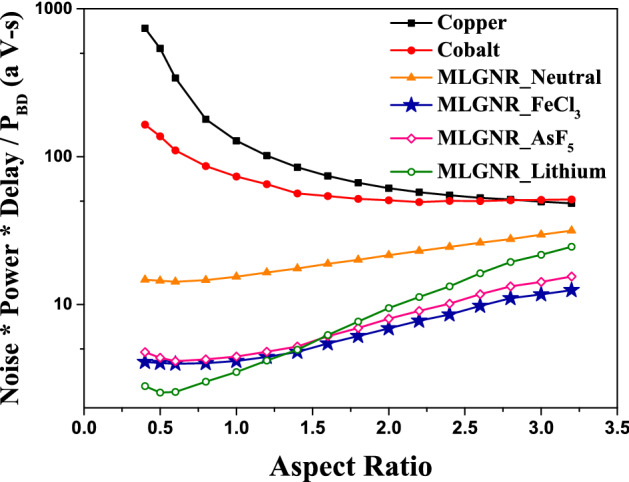
Optimized *AR* at minimum (noise power delay product / breakdown power) for 10 $\mu$m long interconnects.

**Figure 11:**
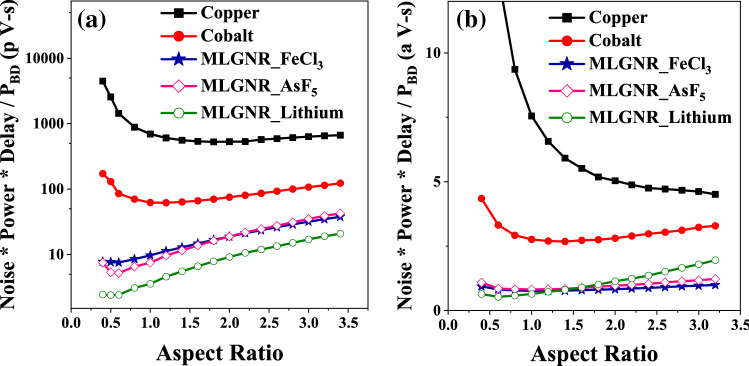
Optimized *AR* at minimum (noise power delay product / breakdown power) in (**a**) global level (L = 1 mm) and (**b**) local level (L = 500 nm) interconnects.

We have considered the metric $$(Noise*Delay*Power/P_{BD})$$ as the Figure of Merit (FOM) which gives a measure of performance, noise, power consumption and reliability effects. The optimization of *AR* by minimizing this FOM for intermediate level interconnects is shown in Fig. 10. Here, we get an optimization AsF_5_, Fecl_3_, Lithium doped MLGNR interconnects at 0.6, 0.6 and 0.5, respectively. The FOM first decreases and then saturates with increasing *AR* for neutral MLGNR. It keeps decreasing with increasing *AR* for cobalt and copper interconnects. Doped MLGNRs are far better candidates considering an overall performance and reliability aspect specially at lower *AR*. Although Lithium dopant gives the highest advantage but Fecl_3_ is explored more in experimental literature’s. Figure 11a,b show the optimization for global and local level interconnects, respectively. FOM for global level AsF_5_, Fecl_3_, Lithium doped MLGNR interconnects are minimum at *AR* of 0.6, 0.6 and 0.5, respectively. And for local level it is 1.0, 1.0 and 0.6, respectively. Doped MLGNR interconnect outperforms all other candidates by having minimum FOM at all metal levels as shown in Figs. 10 and 11. Thus we propose swapping all the metal lines (conventional copper lines) with any doped MLGNR interconnect in order to achieve performance as well as reliability.

### Validation with existing results

**Table 2:** Calibration of our results (FeCl_3_ doped interconnect) with^10^ at 11 nm technology node.

Performance metrics	Results^10^	Our results
Delay ($p-sec$)	5.5	5.236
Energy (x$10^{-16} J$)	2.80	2.648
Power ($\mu W$/mm)	20.8	19.49

**Table 3:** Validation of our numerical model (section “Formulation and methodology”) with^10^ and^14^.

Optimization parameters	Optimized AR (Existing Works)	Optimized AR (Numerical Model)
Minimizing Delay of FeCl_3_ doped MLGNR interconnect	0.8^10^	0.88
Minimizing Delay of AsF_5_ doped TC-MLGNR interconnect	0.958^14^	1.04
Minimizing Delay ofLithium doped TC-MLGNR interconnect	0.802^14^	0.871
Minimizing EDP of AsF_5_ doped TC-MLGNR interconnect	0.479^14^	0.52
Minimizing EDP of *Lithium* doped TC-MLGNR interconnect	0.37^14^	0.415

For calibration of the electrical model, we have estimated delay, energy and power consumption in $FeCl_{3}$ doped MLGNR at 11 nm technology node utilizing the models described in this work (refer “Circuit Modeling of MLGNR Interconnects - An Overview” section) and compared them with results obtained by Jiang et al.^10^. The comparison is shown in Table 2. All the dimensional parameters are taken from^10^ for comparison.

Also to validate our numerical model, comparison is shown in Table 3. Optimized *AR* estimated using our numerical model is compared with that obtained from existing works^10,14^. All the dimensional parameters are considered to be same as in respective papers for a fair comparison.

### Comparison with IRDS roadmap suggestion

Table 4 shows a comparison between our results (considering $FeCl_3$ doped MLGNR interconnect) and results obtained using IRDS roadmap guidelines. Here, minimum delay, *NDP* and *PDP* are calculated and compared considering the optimized *AR* obtained in our work and the *AR* prescribed by IRDS 2018 roadmap^1^. It can be observed that there is an insignificant advantage in intermediate level interconnect performance but when it comes to global level, a substantial improvement is registered. This improvement in global metal line becomes more valuable as the effect of via is more dominant in global metal path. Our results show improved performance in Fecl_3_ doped MLGNR interconnect for optimized value of *AR* as compared to IRDS roadmap 2015 prescribed *AR*. Also the improvement in *FOM* metric is significant indicating lower *AR* should be adopted considering overall performance and reliability.

**Table 4:** Comparison of our results (considering $FeCl_3$ doped MLGNR interconnect) with IRDS 2018 Roadmap suggestion of Aspect Ratio (for copper interconnect)^1^ at 7 nm technology node.

Performance metrics	IRDS specs	Our results	% decrease
**Intermediate level** (L=10 $\mu$m)	(*AR* = 2.1)		
Delay ($p-sec$)	104	102 (optimized *AR* = 1.4)	1.92
NDP ($p V-sec$)	1.75	0.975 (optimized *AR* = 0.8)	44
PDP ($a W-sec$)	3.36	3.07 (optimized *AR* = 1.0)	8.63
PDP/$P_{BD}$ ratio ($f-sec$)	0.46	0.43 (optimized *AR* = 1.0)	6.28
NPDP/$P_{BD}$ ratio ($a V-sec$)	7.765	3.97 (optimized *AR* = 0.6)	48.85
**Global level** (L=1 mm)	(*AR* = 2.3)		
Delay ($n-sec$)	0.531	0.397 (optimized *AR* = 1.0)	25.2
NDP ($n V-sec$)	0.088	0.044 (optimized *AR* = 0.6)	50.45
PDP ($f W-sec$)	0.467	0.243 (optimized *AR* = 0.8)	48
PDP/$P_{BD}$ ratio ($f-sec$)	0.142	0.074 (optimized *AR* = 0.8)	48
NPDP/$P_{BD}$ ratio ($a V-sec$)	23.51	7.541 (optimized *AR* = 0.6)	68

### Impact of contact resistance

Table 5 gives us an understanding of effect of contact resistance on numerically optimized *AR* and FOM. Here we have varied the contact resistance from 5 $K\Omega$ to 20 $K\Omega$^25^. As we can see, intermediate and global lines are not affected by it. But when it comes to local lines, increase in contact resistance leads to significant decrease in performance and increase in optimized *AR*. With a 75% increase in contact resistance, $\sim$66%, $\sim$5.5% and $\sim$0.4% degradation in FOM of local, intermediate and global level Fecl_3_ doped MLGNR interconnect, respectively is witnessed. Optimized *AR* also experiences an increase of $\sim$26%, $\sim$2.5% and $\sim$1.8% in local, intermediate and global level, respectively. This infers that when the contact resistance increases, then in order to compensate the decrease in performance, *AR* can be increased (which will lead to increase in number of layers and thus decrease in contact resistance). So the performance of MLGNR interconnects will not improve beyond a certain limit.

**Table 5:** Impact of contact resistance on optimized *AR* and FOM Fecl_3_ doped MLGNR interconnects at 7 nm technology node.

Contact resistance	Optimized *AR* (FOM value)		
	Local	Intermediate	Global
5 $K\Omega$	0.87 (0.567 aV-sec)	0.617 (3.97 aV-sec)	0.58 (7.541 pV-sec)
10 $K\Omega$	1.02 (0.765 aV-sec)	0.62 (4.01 aV-sec)	0.582 (7.547 pV-sec)
15 $K\Omega$	1.09 (1.07 aV-sec)	0.626 (4.07 aV-sec)	0.586 (7.556 pV-sec)
20 $K\Omega$	1.18 (1.8 aV-sec)	0.633 (4.2 aV-sec)	0.591 (7.57 pV-sec)

#### Impact of scaling

**Figure 12:**
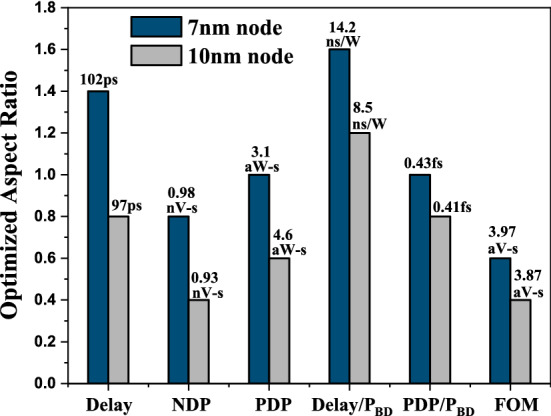
Impact of scaling on Fecl_3_ doped MLGNR interconnect of length 10 $\mu$m (Intermediate level).

**Figure 13:**
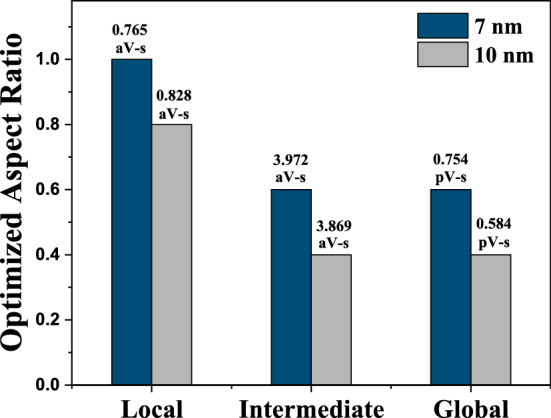
Impact of scaling on Fecl_3_ doped MLGNR interconnect considering FOM at local, intermediate and global level.

The Impact of scaling (from 10 to 7 nm node) on optimized *AR* value of 10 $\mu$m long Fecl_3_ doped MLGNR interconnect is shown in Fig. 12. Here, we can observe that scaling leads to an increase in optimized *AR* value along with degradation in performance and reliability for intermediate level interconnects following IRDS trends. When compared to copper with cobalt liner (considering IRDS suggested dimensions), Improvement in FOM of Fecl_3_ doped MLGNR interconnect (calculated at optimized *AR*) is $\sim 54\%$, $\sim 93\%$ and $\sim 98\%$ at local, intermediate and global levels, respectively for 7 *nm* and for 10 nm, it is $\sim 43\%$, $\sim 85\%$ and $\sim 91\%$ at local, intermediate and global levels, respectively. The point to be noted here is that even though the performance of MLGNR degrades with scaling but when compared to copper, the performance increases with decreasing technology node. Figure 13 gives an understanding on effect of scaling on local, intermediate and global level Fecl_3_ doped MLGNR interconnect considering the FOM. Scaling down from 10 *nm* to 7 *nm* node leads to degradation in FOM in Fecl_3_ doped MLGNR interconnect by 2.6% and 22.6% in intermediate and global level, respectively even though we increase the *AR* from 0.4 to 0.6. But in case of local level, FOM is improved while scaling down by 8% if we increase the *AR* from 0.8 to 1.0. It is evident that with scaling there is a need of increase in *AR* in order to improve performance and reliability. The optimized (recommended) aspect ratio in this paper is less than 1.0. Although scaling down from 10 to 7 nm leads to an increase in optimized *AR*, but still it is lower as compared to IRDS suggestions (*AR* > 2). Jiang et al. have fabricated Fecl_3_ doped MLGNR interconnect with aspect ratio of 0.4 and 0.6^10^, which strengthens our claim from manufacturing point of view.

## Formulation and methodology

**Figure 14:**
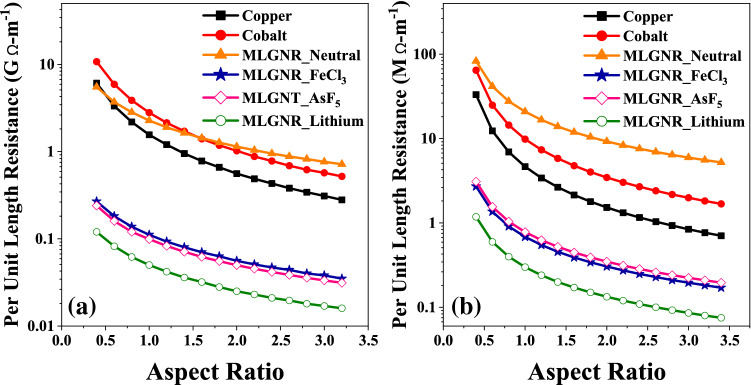
Per unit length electrical resistance for (**a**) intermediate level (L = 10 $\mu$m) and (**b**) global level (L = 1 mm) interconnects.

This proposed methodology provides a detailed numerical dimensional optimization procedure and is applicable to any generic nano-interconnect system. This numerical methodology can avoid costly simulators set-ups and expensive fabrication procedures for providing the dimensional design guidelines to achieve such improvement in performance. Aspect ratio (*AR*) optimization serves to be an effective dimensional optimization technique for achieving superior performance and reliability metrics. Here, dependence of the RLC parameters on *AR* is discussed in order to establish relation between *AR* of interconnect and its delay, noise induced effects, power consumption and breakdown power.

From $N_{oL}=1+\lfloor \frac{t}{\delta }\rfloor$, we get $N_{oL} \propto AR$. And $N_{ch}$ is a function of width of the interconnect. Therefore $R_{qtm} \propto (1/AR)$ as $N_{ch}$ is constant and $N_{oL}$ is a function of *AR*.

So, $R_{pul} \propto (1/AR)$ as shown in Fig. 14 because $G_{pul}$ is a constant here as it is a function of width and $N_{oL}$ is directly proportional to *AR*.

Similarly, $C_{pul}$ is an increasing function of *AR*. $C_{gnd}$, $C_{intra}$ and $C_{inter}$ collectively adds to $C_{elc}$, where $C_{inter}$ can be neglected^15^. They can be described as functions of *AR* as mentioned in Eqs. (7) and (8),

7 $$\begin{aligned}&C_{gnd}\propto \left[ \frac{1}{AR}+\left( \frac{1}{1+AR}\right) ^{1.16}+1\right] \end{aligned}$$

8 $$\begin{aligned}&C_{intra}\propto \Bigg [A.R^{1.7}\left( \frac{1}{1+AR}\right) ^{0.7}+1 \left( \frac{AR}{1+AR}\right) ^{0.8}+1\left( \frac{AR}{1+AR}\right) ^{3.5}\Bigg ] \end{aligned}$$

The dependence of $C_{cup}$ on *AR* is described as,

9 $$\begin{aligned} C_{cup}\propto \Biggl [ AR^{1.09}\left( \frac{1}{1+AR}\right) ^{0.09}+1\left( \frac{AR}{1+AR} \right) ^{1.18}+1\Biggr ] \end{aligned}$$

Noise and power consumption are a function of capacitance and hence they increase with increasing *AR*. However, delay is the dominating factor in *NDP* and *PDP* metrics. So, the expression of *NDP* and *PDP* leads to an optimized *AR* value.

### Delay centric design

Propagation delay in an interconnect is basically a function of its *RC* product. With increasing *AR*, resistance decreases as shown in Fig. 14. But the capacitance increases, therefore an optimized value of *AR* is obtained for minimum delay point. The transfer function for the crosstalk delay or noise evaluation in the victim net will be denoted as *H*(*s*). The second-order $Pade's$ expansion of the transfer function is given by^16^:

10 $$\begin{aligned} H(s)\approx {\frac{1}{1+sb_{1}+s^{2}b_{2}}} \end{aligned}$$

The two poles of the transfer function are:

11 $$\begin{aligned} s_{1,2}={\frac{-b_{1}\pm \sqrt{b_{1}^{2}-4b_{2}}}{2b_{2}}} \end{aligned}$$

where $b_1$ and $b_2$ are functions of R,L,Cs provided in^16^. They can be defined as a function of *AR* as follows,

12 $$\begin{aligned}&b_1 \propto \frac{c_1}{AR}+c_2+c_3A.R \end{aligned}$$

13 $$\begin{aligned}&b_2 \propto \frac{a_1}{AR}+a_2 \end{aligned}$$

The coefficients $a_1$, $a_2$, $c_1$, $c_2$, $c_3$ are described as:

14 $$\begin{aligned}&a_1 = \frac{2R_dC_dC_lR_{qtm}\delta +LC_lL_{qtm}\delta +2LC_lR_{qtm}^2C_{qtm} \delta }{N_{ch}w} + \frac{LC_l\delta R_{qtm}C_{qtm}}{G_{pul}w} + \frac{L^3C_lC_{qtm}N_{ch}\delta }{6G_{pul}^2w} \end{aligned}$$

15 $$\begin{aligned}a_2 &= \frac{L^2L_{qtm}C_{qtm}}{2} + \frac{L^4C_{qtm}^2N_{ch}^2}{24.G_{pul}^2} + \frac{L^2R_d(C_d+C_l)C_{qtm}N_{ch}}{2G_{pul}} + \frac{L^3R_{qtm}C_{qtm}^2N_{ch}}{6G_{pul}} \nonumber \\ &\quad + LR_d(C_d+C_l)R_{qtm}C_{qtm} + \frac{L^3R_dC_{qtm}^2N_{ch}^2w}{6G_{pul}\delta } \end{aligned}$$

16 $$\begin{aligned}&c_1 = \frac{2C_lR_{qtm}\delta }{N_{ch}w} + \frac{LC_l\delta }{G_{pul}w} \end{aligned}$$

17 $$\begin{aligned}&c_2 = LR_{qtm}C_{qtm} + \frac{L^2C_{qtm}N_{ch}}{2G_{pul}} + R_d(C_d+C_l) \end{aligned}$$

18 $$\begin{aligned}&c_3 = \frac{LR_dC_{qtm}N_{ch}w}{\delta } \end{aligned}$$

The step response, which is the inverse Laplace transform of $\frac{1}{H(s)}$, is given by:

19 $$\begin{aligned} v(t)=V_{0}\left[ \frac{1-{s_{2}}}{s_{2}-s_{1}}\exp (s_{1}t) +\frac{{s_{1}}}{s_{2}-s_{1}}\exp (s_{2}t)\right] \end{aligned}$$

The 50% propagation delay ($\tau$) is given by^16^,

20 $$\begin{aligned}0.5-{\frac{s_{2}}{s_{2}-s_{1}}}\exp (s_{1}\tau ) +\frac{{s_{1}}{s_{2}-s_{1}}}{\exp }(s_{2}\tau ) = 0 \end{aligned}$$

21 $$\begin{aligned}&\tau =\frac{b_2}{b_1}\left[ ln\left( 0.5\right) +ln\left( \frac{b_1}{\sqrt{b_1^2-4b_2}}-1\right) -ln\left( \frac{b_1}{\sqrt{b_1^2-4b_2}} +1\right) \right] \end{aligned}$$

Delay in terms of AR can be defined as:

22 $$\begin{aligned} \begin{aligned} \tau&=\left( \frac{a_1+a_2.AR}{c_1+c_2.AR+c_3.AR^2}\right) \Biggl [ln\left( 0.5\right) +ln\left( \frac{c_1+c_2.AR+c_3.AR^2}{\sqrt{q_1+q_2.AR+q_3.AR^2+q_4.AR^3+q_5.AR^4}}-1\right) \\& \quad -ln\left( \frac{c_1+c_2.AR+c_3.AR^2}{\sqrt{q_1+q_2.AR+q_3.AR^2 +q_4.AR^3+q_5.AR^4}}+1\right) \Biggr ] \end{aligned} \end{aligned}$$

Setting the derivative of delay with respect to *AR* to zero, we can obtain the optimized *AR* at which delay is minimum:

23 $$\begin{aligned}&{\frac{\partial \tau }{\partial AR}}=0 \end{aligned}$$

24 $$\begin{aligned}&\tau =u*v \implies {\frac{\partial \tau }{\partial AR}} = u*{\frac{\partial v}{\partial AR}} + v*{\frac{\partial u}{\partial AR}} = 0 \end{aligned}$$

where $u,v, {\frac{\partial u}{\partial AR}}$ and ${\frac{\partial v}{\partial AR}}$ are described as:

25 $$\begin{aligned}&u = \left( \frac{a_1+a_2.AR}{c_1+c_2.AR+c_3.AR^2}\right) \end{aligned}$$

26 $$\begin{aligned}v &= \left[ ln\left( 0.5\right) +ln\left( \frac{c_1+c_2.AR+c_3.AR^2}{\sqrt{q_1+q_2.AR+q_3.AR^2+q_4.AR^3+q_5.AR^4}}-1\right) \right. \nonumber \\&\quad \left. -ln\left( \frac{c_1+c_2.AR+c_3.AR^2}{\sqrt{q_1+q_2.AR+q_3.AR^2 +q_4.AR^3+q_5.AR^4}}+1\right) \right] \end{aligned}$$

27 $$\begin{aligned}&{\frac{\partial u}{\partial AR}} = \left[ \frac{a_2(c_1+c_2.AR+c_3.AR^2)-(a_1+a_2.AR)(c_2+2c_3.AR)}{(c_1+c_2.AR+c_3.AR^2)^2}\right] \end{aligned}$$

28 $$\begin{aligned}{\frac{\partial v}{\partial AR}} &= \left( \sqrt{q_1+q_2.AR+q_3.AR^2 +q_4.AR^3+q_5.AR^4}.(c_2+2c_3.AR)\right) \nonumber \\&\quad -\left[ (c_1+c_2.AR+c_3.AR^2)\left( \frac{q_2+2q_3.AR+3q_4.AR^2 +4q_5.AR^3}{2\sqrt{q_1+q_2.AR+q_3.AR^2+q_4.AR^3+q_5.AR^4}}\right) \right] \end{aligned}$$

The constant coefficients $q_1$, $q_2$, $q_3$, $q_4$, $q_5$ are described here as follows,

29 $$\begin{aligned} q_1 &= {} c_2^2 \end{aligned}$$

30 $$\begin{aligned} q_2 &= {} 2c_1c_2-4a_2 \end{aligned}$$

31 $$\begin{aligned} q_3 &= {} c_1^2+2c_2c_3-4a_1 \end{aligned}$$

32 $$\begin{aligned} q_4 &= {} 2c_1c_3 \end{aligned}$$

33 $$\begin{aligned} q_5 &= {} c_3^2 \end{aligned}$$

Equation (23) can be numerically solved (here Newton Raphson is used) to obtain the optimized *AR* value at which delay is minimum. The initial guess value of *AR* was taken as 1 and the equation converged in less than 8 iterations giving optimized *AR* value of 1.418, 1.3329 and 1.213 for intermediate level AsF_5_, FeCl_3_ and Lithium doped MLGNR interconnects, respectively. Global level AsF_5_, FeCl_3_, and Lithium doped MLGNR interconnects optimized at *AR* of 1.06, 0.87 and 0.76, respectively.

### FOM centric design

Similar approach is adopted to obtain the optimized *AR* at minimum FOM. We define Figure of Merit (FOM) as:

34 $$\begin{aligned} FOM=\tau *N_{peak}*Power/P_{BD} \end{aligned}$$

where $\tau$ is given in equation (4), peak noise voltage ($N_{peak}$) is given by^17,18^,

35 $$\begin{aligned} N_{peak}= \frac{C_{cup}}{s_2}\left( \frac{s_2}{s_1-s_2}\right) \left[ \left( \frac{s_1}{s_2}\right) ^{\frac{-s_2}{(s_1-s_2)}} -\left( \frac{s_1}{s_2}\right) ^{\frac{-s_1}{(s_1-s_2)}}\right] \end{aligned}$$

The total power consumed in the interconnect is mainly because of the power consumed by driver and load buffers which is given by^19^,

36 $$\begin{aligned} P_{total}=(N_{opt}+2)(P_{switch}+P_{short}+P_{leak}) \end{aligned}$$

where $P_{switch}$, $P_{short}$ and $P_{leak}$ are switching, short circuit and leakage power of a repeater, respectively. The definition of various parameters are specified in detail in^20^. Switching power dominates the equation thus is considered for further calculation for simplicity^19^.

37 $$\begin{aligned} P_{ \mathrm{switching}}=S_f (S_r(c_{d}+c_{l})+L_{rep}C_{pul})V_{ {DD}}^{2}f_{ \mathrm{clk}} \end{aligned}$$

where $V_{DD}$ is power supply voltage, $f_{clk}$ is the clock frequency, $L_{rep}$ is the inter repeater stage length, $S_r$ is the ratio of buffer size to minimum sized buffer and $S_f$ is the switching factor, which is the fraction of repeaters on a chip that are switched during an average clock cycle. It can be taken as 0.15^20^. $P_{switch}$ as a function of *AR* can be defined as:

38 $$\begin{aligned} \begin{aligned} P_{switch}&=\left[ \frac{1}{AR}+\left( \frac{1}{1+AR}\right) ^{1.16} +AR\left( \frac{AR}{1+AR}\right) ^{0.7}+\left( \frac{AR}{1+AR}\right) ^{0.8} +\left( \frac{AR}{1+AR}\right) ^{3.5}\right] \\&\left[ \sqrt{AR^2+AR(1+AR)^{1.16}+AR+(1+AR)^{0.8}+\left( \frac{(1+AR)}{AR}\right) ^{0.7}+(1+AR)^{3.5}}+1\right] \end{aligned} \end{aligned}$$

In order to have an understanding of reliability, we need to calculate and analyse the power consumed at the point where GNR interconnects breakdown and is given by^9,21^,

39 $$\begin{aligned} P_{BD}=gL(T_B-T_A) \Biggl [\frac{cosh(\frac{L}{2L_{TH}})+gL_{TH}R_T sinh(\frac{L}{2L_{TH}})}{cosh(\frac{L}{2L_{TH}})+gL_{TH}R_T sinh(\frac{L}{2L_{TH}})-1}\Biggr ] \end{aligned}$$

where $L_{TH}$ is defined as the thermal healing length of the metal line^21^. Breakdown power as a function of *AR* can be defined as:

40 $$\begin{aligned} P_{BD} \propto \Biggl [\frac{cosh(\frac{1}{\sqrt{AR}})+\sqrt{AR}sinh(\frac{1}{\sqrt{AR}})}{cosh(\frac{1}{\sqrt{AR}})+\sqrt{AR}sinh (\frac{1}{\sqrt{AR}})-1}\Biggr ] \end{aligned}$$

Setting the derivative of FOM with respect to *AR* to zero, we can obtain the optimized *AR* at which FOM is minimum:

41 $$\begin{aligned} {\frac{\partial FOM}{\partial AR}}=0 = \frac{P_{BD}\left( {\frac{\partial \tau }{\partial AR}}N_{peak}P_{sw}+{\frac{\partial N_{peak}}{\partial AR}}\tau P_{sw}+{\frac{\partial P_{sw}}{\partial AR}}\tau N_{peak}\right) -\tau N_{peak} P_{switch} {\frac{\partial P_{BD}}{\partial AR}}}{(P_{BD})^2} \end{aligned}$$

This equation is numerically solved (here Newton Raphson is used) to obtain optimized *AR* value which minimizes the FOM. Here the initial guess value of *AR* was taken as 0.5 and the equation converged in 11 iterations giving optimized *AR* value of 0.633, 0.61 and 0.583 for intermediate level AsF_5_, FeCl_3_, and Lithium doped MLGNR interconnects, respectively. Global level AsF_5_, FeCl_3_, and Lithium doped MLGNR interconnects optimized at *AR* of 0.585, 0.579 and 0.481, respectively. And local level AsF_5_, FeCl_3_, and Lithium doped MLGNR interconnects optimized at *AR* of 1.08, 0.987 and 0.623, respectively.

## Conclusion

This work focuses on numerically determining optimum aspect ratio in order to improve performance, reliability and minimize noise effects and power consumption. This approach will be extremely useful to industry for selection of *AR* of interconnects as it is a non-SPICE method. Our approach provides a detailed guideline for the Aspect ratio optimization and reduces fabricational cost to achieve high performance and reliability MLGNR interconnects by reducing iterations during fabrication process for achieving desired performance. The optimized *AR* of AsF_5_, FeCl_3_, Lithium doped MLGNR interconnects by minimizing delay is obtained at 1.4, 1.4 and 1.2 for intermediate level and 1.0, 1.0 and 0.8 for global level interconnects, respectively. Intermediate level, AsF_5_, FeCl_3_, Lithium doped MLGNR interconnects have an optimized *AR* of 1.0, 1.0 and 0.6 and global levels have an optimized *AR* of 0.8 at which minimum *PDP* is registered. $PDP/P_{BD}$ ratio is minimized at an aspect ratio of 1.0, 1.0 and 0.6, respectively. The FOM is minimized at an aspect ratio of 1.0, 1.0 and 0.6 for local level and 0.6, 0.6 and 0.5 for intermediate and global level AsF_5_, FeCl_3_, Lithium doped MLGNR interconnects, respectively. Increase in contact resistance leads to significant decrease in performance and increase in optimized *AR* of local FeCl_3_ doped MLGNR interconnect. As we scale down, the optimized *AR* increases with decrease in performance and reliability for intermediate and global levels. But while scaling down, increase in optimized *AR* leads to better FOM in local level doped MLGNR interconnect. When compared to copper (considering IRDS suggested dimensions), Improvement in FOM of FeCl_3_ doped MLGNR interconnect (calculated at optimized *AR*) is $\sim 54\%$, $\sim 93\%$ and $\sim 98\%$ at local, intermediate and global levels, respectively for 7 nm and for 10 nm, it is $\sim 43\%$, $\sim 85\%$ and $\sim 91\%$ at local, intermediate and global levels, respectively. When compared to IRDS suggestion, the estimated delay in intermediate level FeCl_3_ doped MLGNR interconnect is improved by $\sim$2%, *NDP* by 44%, *PDP* by $\sim$9%, the $PDP/P_{BD}$ is improved by $\sim$6% and FOM by $\sim$49%. Similarly in global level, delay, *NDP*, *PDP*, $PDP/P_{BD}$ and FOM is improved by 25%, 50%, 48%, 48% and 68%, respectively. This study has systematically formulated a numerical optimization methodology and guideline for selecting an optimized aspect ratio to achieve improved performance and reliability for doped MLGNR interconnects.
